# Gene copy number variation in natural populations of *Plasmodium falciparum* in Eastern Africa

**DOI:** 10.1186/s12864-018-4689-7

**Published:** 2018-05-21

**Authors:** Joan Simam, Martin Rono, Joyce Ngoi, Mary Nyonda, Sachel Mok, Kevin Marsh, Zbynek Bozdech, Margaret Mackinnon

**Affiliations:** 1 KEMRI-Wellcome Trust Research Program, Kilifi, Kenya; 2 Department of Microbiology and Molecular Medicine, Medical Faculty, University of Geneva, Geneva, Switzerland; 3 Department of Microbiology and Immunology, Columbia University, New York, USA; 4 Nuffield Department of Medicine, University of Oxford, Oxford, UK; 5 School of Biological Sciences, Nanyang Technological University, Singapore, Singapore; 6 http://www.researcherid.com/rid/L-3155-2013; 7 Independent Researcher, Nairobi, Kenya

**Keywords:** Copy number variation, *Plasmodium falciparum*, Adaptation

## Abstract

**Background:**

Gene copy number variants (CNVs), which consist of deletions and amplifications of single or sets of contiguous genes, contribute to the great diversity in the *Plasmodium falciparum* genome. In vitro studies in the laboratory have revealed their important role in parasite fitness phenotypes such as red cell invasion, transmissibility and cytoadherence. Studies of natural parasite populations indicate that CNVs are also common in the field and thus may facilitate adaptation of the parasite to its local environment.

**Results:**

In a survey of 183 fresh field isolates from three populations in Eastern Africa with different malaria transmission intensities, we identified 94 CNV loci using microarrays. All CNVs had low population frequencies (minor allele frequency < 5%) but each parasite isolate carried an average of 8 CNVs. Nine CNVs showed high levels of population differentiation (F_ST_ > 0.3) and nine exhibited significant clines in population frequency across a gradient in transmission intensity. The clearest example of this was a large deletion on chromosome 9 previously reported only in laboratory-adapted isolates. This deletion was present in 33% of isolates from a population with low and highly seasonal malaria transmission, and in < 9% of isolates from populations with higher transmission. Subsets of CNVs were strongly correlated in their population frequencies, implying co-selection.

**Conclusions:**

These results support the hypothesis that CNVs are the target of selection in natural populations of *P. falciparum*. Their environment-specific patterns observed here imply an important role for them in conferring adaptability to the parasite thus enabling it to persist in its highly diverse ecological environment.

**Electronic supplementary material:**

The online version of this article (10.1186/s12864-018-4689-7) contains supplementary material, which is available to authorized users.

## Background

*P. falciparum*, the most virulent of the species that cause malaria in humans, is characterized by extensive genetic diversity that enables the parasite to escape host immune defence, resist antimalarial drugs and pose a further challenge to vaccine development [1–4]. Sources of genomic variation in this parasite range from changes at the single nucleotide level through to large structural alterations of the chromosomes. Gene copy number variants (CNVs) lie between these extremes, consisting of deletions and amplifications of a gene or set of contiguous genes. CNVs are thought to directly affect the level of gene expression through altering gene dosage, but also indirectly through modification of the chromatin environment in the vicinity of the CNV (reviewed in [5]). Potentially, therefore, CNVs may influence clinically relevant parasite phenotypes such as drug resistance, erythrocyte invasion and transmissibility.

Interest in CNVs in malaria parasites has been driven by confirmation of their role in adaptation, evolution and disease in other organisms [6–9], boosted by advances in technologies for high-throughput genome-wide scans of the malaria genome [10]. Surveys of parasite lines adapted to in vitro culture conditions in the laboratory, both long-term [11–17] and short-term [18], have revealed many CNVs in the *P. falciparum* genome. Common among these are two large deletions that abrogate traits which are crucial to survival in vivo but dispensable in vitro. These are the deletion of a region on chromosome 9 that contains several genes required for formation of gametocytes, the life stage required for transmission to new hosts via mosquitoes [19, 20], and a region on chromosome 2 containing a gene encoding the knob associated histidine rich protein (KAHRP) that mediates binding of the infected red blood cell to other host cells (cytoadherence) thereby allowing the parasite to avoid circulation through the spleen where it would otherwise be destroyed [21]. Another example of an in vitro-associated CNV is the amplification of reticulocyte-binding protein 1 encoding gene (*rh1*) [12, 16, 18, 22]. This protein is involved in red cell invasion [23] and appears to be associated with increased parasite asexual replication rate in vitro [16, 22]. In vitro selection for drug resistance has uncovered further CNVs. Examples include amplification of genes encoding multi-drug resistance protein 1 (*Pfmdr1*) that associates with resistance to multiple drugs in in vitro studies [24]; amplifications in the genes encoding the cysteine proteases falcipain 2 (*FP2a* and *FP*2*b*) and falcipain 3 (*FP3*) in which mutations have been associated with resistance to the antimalarial compound artemisinin [25], and which help breakdown haemoglobin in the food vacuole [26], a process that is required for artemisinin to be effective [27]; a deletion of 15 consecutive genes on chromosome 10 in parasites bearing mutations in the chloroquine resistance transporter gene (*Pfcrt*) [28]; deletion of 23 adjacent genes in chromosome 14 in strains resistant to the anti-malarial compound, fosmidomycin [13]; and amplification of the gene encoding GTP cyclohydrolase 1 (*gch1*) [12, 18], an enzyme high in the folate synthesis pathway and thus a potential target for the antifolate class of anti-malarial drugs. Most of these laboratory-derived CNVs have been shown to affect expression levels of genes inside the CNV and, in a few cases, genes located on other chromosomes [18, 28]. Combined, the evidence from in vitro studies strongly supports the hypothesis that CNVs play an important role in parasite adaptation to novel environments.

The relevance of in vitro-based studies of CNVs to parasite adaptation in the field remains unclear, however. For example, the cytoadherence and gametocyte-linked deletions on chromosome 2 and 9, and the replication-linked *rh1* amplification have not been found among the limited number of field isolates of *P. falciparum* surveyed to date [22, 29]. This implies strong selection against these mutations in vivo. On the other hand, CNVs involving drug resistance have been observed in the field, e.g., amplifications in *mdr1* in patients with failed response to drugs [30], and *gch1* amplification in populations subjected to antifolate drug pressure [31, 32], thus reflecting their adaptive value under field conditions if there are novel selection forces at play. Many CNVs not observed among laboratory isolates and of unknown clinical or adaptive significance have been discovered in global surveys of field populations [29]. Indeed, it is estimated that between 0.3 - 6% of the parasite’s genome is subject to variation in gene copy number. This is greater than the fraction represented by single nucleotide polymorphisms (SNPs).

Thus the evidence to date suggests that CNVs play a significant role in adaptation of the parasite to novel environmental conditions. Whether this includes naturally varying factors such as immunity, mosquito density and host genetics, as distinct from selective agents not previously encountered such as drugs, remains unknown. Here, we test the hypothesis that CNVs provide the source of adaptive variation used by the parasite to evolve in response to natural environmental variation. Empirical support for this hypothesis would have implications for malaria control programmes that change the epidemiological setting of the parasite. We examine this hypothesis by analysing CNV variation among geographically and temporally separated populations of *P. falciparum* in Eastern Africa that differ widely in malaria transmission intensity and thus related selection pressures. We further test for experimental sources of variation in the detectability of CNVs in order to account for or rule out experimental bias in our results.

## Results and discussion

### General properties of CNVs

From 183 *P. falciparum* infected blood samples (Table 1), using a microarray previously validated for CNV detection [18] and after applying stringent CNV definition criteria, a total of 94 different CNVs with minor allele frequency (MAF) greater than 2.2% (i.e., found in 4 or more samples), and containing 228 different genes, were detected (Additional file 1). Thirty-one of these were classed as deletions, 58 as amplifications and 5 as carrying both types of alleles (“amp-dels”). These classifications were made in reference to the P4 isolate [18] with one exception, namely, cnv9_269 for which P4 carried a deletion: in this case, the deletion was defined with respect to the majority of isolates in the sample population.

**Table 1:** Characteristics of the four study populations

Population	Kisumu	Kilifi pre malaria decline	Kilifi post malaria decline	Sudan
Malaria transmission intensity	High	Medium-high	Medium-low	Low
Number of samples	49	33	49	52
Year of sample collection	2008	1994–1996	2010	2007
Median age in months	36	30	53.5	84
(range)	(6–72)	(11–37)	(14–147)	(12–612)
log_10_ median parasitaemia	5.3	4.7	5.1	5.1
(par/μl) (range)	(4.9–5.8)	(4.0–5.8)	(2.5–6.1)	(4.4–5.8)
Median hemoglobin (g/dl)	9.9	9.4	10.6	9.6
(range)	(5.2–15.2)	(5.3–13.2)	(3.4–12.1)	(3.2–12.9)
Median number of clones	2	2	2	2
(range)	(1–6)	(1–7)	(1–5)	(1–5)
Monoclonal infections (percentage)	10.2	33.3	18.4	26.9

**Fig. 1:**
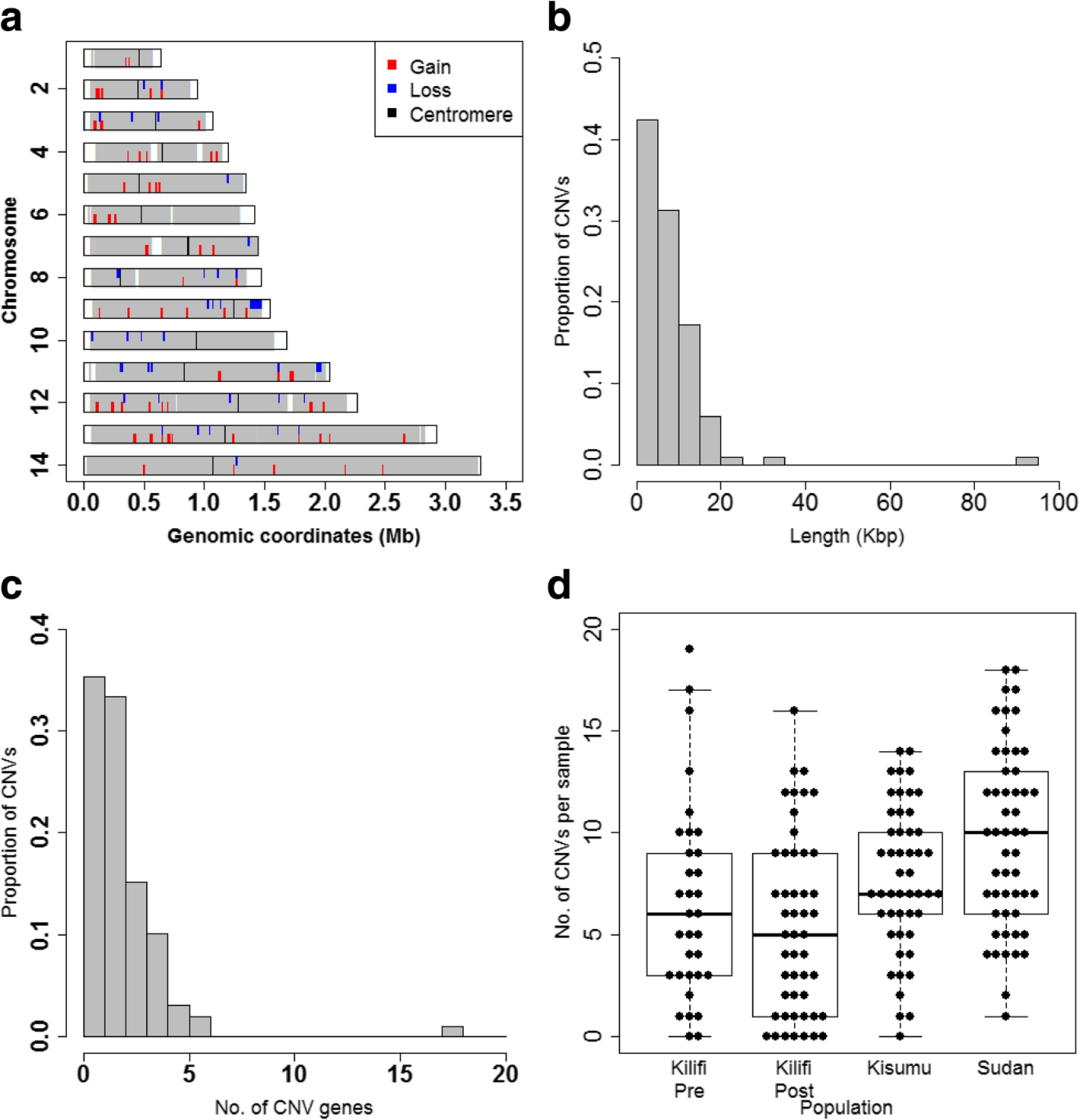
Location and properties of CNVs in the *P. falciparum* genome. **a** Chromosomal location of the 94 CNVs in the 14 nuclear chromosomes of the *P. falciparum* genome (deletions in blue and amplifications in red). White vertical bars represent regions not targeted by the microarray probes. Black vertical bars are locations of centromeres. Distributions of length of CNVs (**b**), number of genes per CNV (**c**) and number of CNVs per sample (**d**) split by study population (horizontal line, median number; top and bottom boundaries, 75th and 25th percentiles; whiskers, minimum and maximum)

CNVs were distributed throughout the 14 chromosomes of the parasite’s nuclear genome (Fig. 1a). CNVs varied in size from 400 bp to 90 kb (Fig. 1b). The majority of CNVs contained less than 3 genes (median of 2) with the largest CNV on chromosome 9 consisting of 18 genes (Fig. 1c). The number of CNVs per sample ranged from 0 to 19 with an average of 8 CNVs per isolate (Fig. 1d) The summed length of all CNVs identified here was 786.7kbp which represents 3.4% of the parasite genome and approximately 4.5% of genes in the genome. Twenty of the 94 CNVs detected here (21%) have been reported in previous studies (Additional file 1), albeit with different breakpoints in some cases: thus the majority of CNVs identified here are novel. Nonetheless, these results accord with previous studies showing considerable amounts of standing variation in CNV loci in field populations [16, 29] and thus support the hypothesis that CNVs play an adaptive role in natural populations of *P. falciparum*.

### Systematic effects on general CNV prevalence

**Fig. 2:**
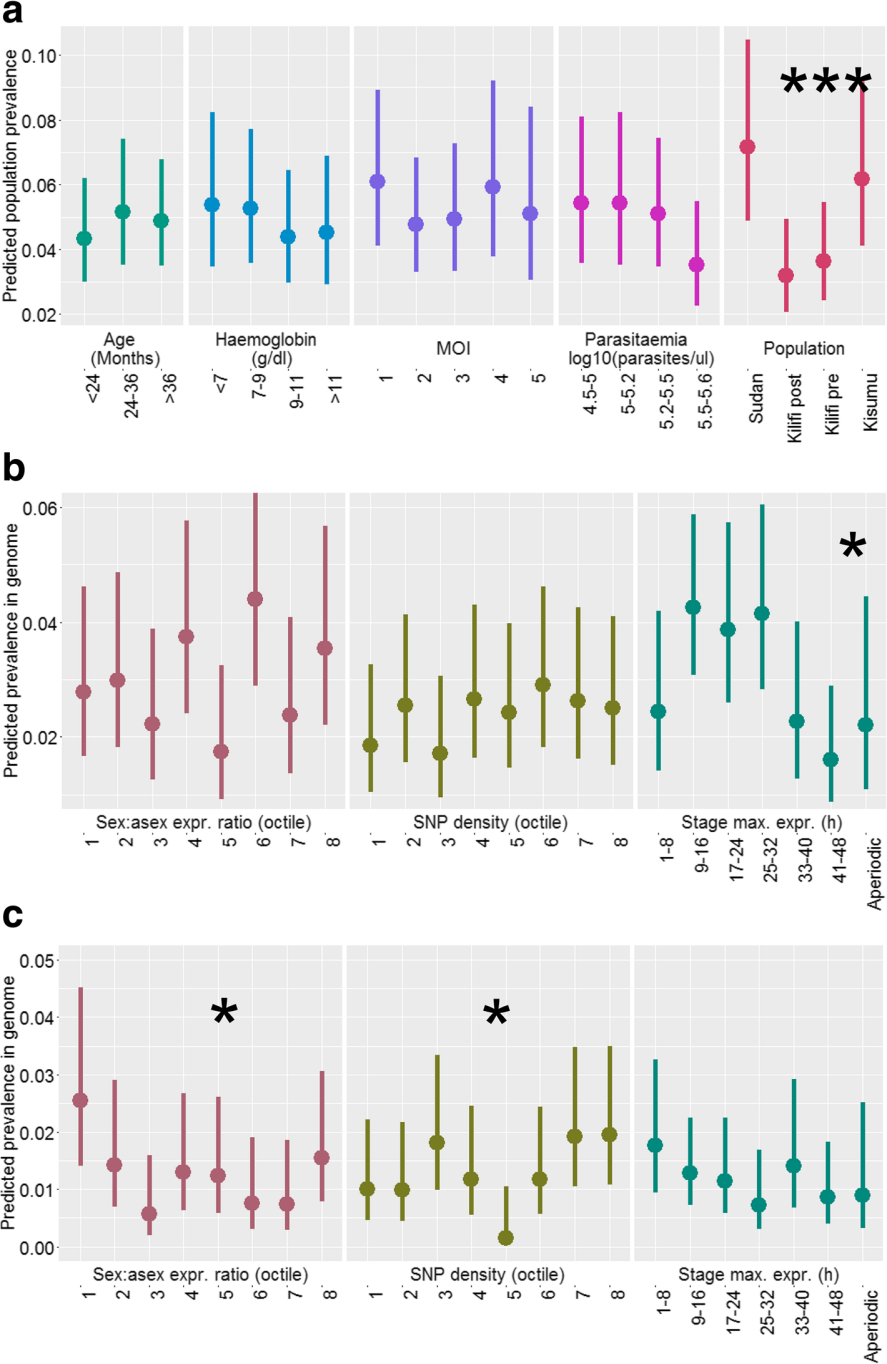
Systematic effects of host, parasite and gene factors on overall CNV prevalence. **a** Effects of host status, infection status and population on population prevalence of all CNVs. **b** Effects of gene properties on genomic prevalence of amplification CNVs. **c** As for b but for deletion CNVs. Points show least-squares means for each level of the factors (x-axis) adjusted for other factors in the model (separate panels). Vertical lines show upper and lower 95% confidence intervals. Significance of each factor is indicated at the top of each panel. *, *P* < 0.05; ***, *P* < 0.001

Most CNVs had low population frequencies (< 5%, Fig. 2). There were no significant effects of multiplicity of infection (MOI), parasitaemia and patient characteristics (age and haemoglobin), or two-way interactions between these factors, on the population prevalence of CNVs overall (*P* > 0.05 by F-test, Fig. 2a). This rules out possible bias in detectability of CNVs due to ‘dilution’ in the case of MOI, and total DNA concentration effects in the case of parasitaemia. By contrast, study population was a strong determinant of overall CNV prevalence (*P* < 0.001), with overall lower prevalence in the medium transmission populations (Kilifi) than in the high and low transmission populations (*P* = 0.35 fitting a linear covariate for transmission intensity, Fig. 2a). Thus population differences in CNV prevalence were not due to bias in detectability caused by sample processing or infection and host-related factors.

Properties of the genes contained within the CNVs and their encoded proteins did not, in general, relate to the probability of being copy number variable. Exceptions to this were as follows: amplification CNVs were more likely to occur in genes that had maximum expression levels during the rings and trophozoite stages within the 48 h replication cycle (*P* = 0.02 by likelihood ratio test, Fig. 2b); deletion CNVs were more prevalent in genes with high asexual:sexual stage expression ratios (*P* = 0.02, Fig. 2c); and there was a significant, but not unidirectional, effect of SNP density on the prevalence of deletion CNVs (*P* = 0.02).

Seven of 255 functional categories of genes were highly significantly enriched for genes belonging to CNVs, five of them for deletion CNVs (nominal *P* < 0.01 by hypergeometric test, only two of which were significant (*P* < 0.05) after accounting for multiple testing (Table 2). Enriched pathways included those involved in export of proteins to the surface of infected red blood cells, and in core metabolic processes such as glycolysis, intracellular trafficking and transcriptional regulation (Table 2). These results indicate that CNVs are not confined to non-essential, non-central processes, as might be expected if the alterations in gene copy number led to dramatic, irreversible changes to gene expression levels.

**Table 2:** Functional gene categories showing significant enrichment for CNVs

Functional gene set	No. of genes in CNVs	Total no. of genes in gene set	Nominal	Adjusted
			*P*-value^a^	*P*-value^b^
Deletions				
Exported proteins - PHISTs	8	48	< 0.001	< 0.001
Characteristics of *Plasmodium falciparum* export proteins that remodel infected erythrocyte	5	35	< 0.001	0.043
Glycolysis	4	27	0.001	0.10
Exported proteins - Unique	6	72	0.0025	0.10
Utilization of phospholipids	4	40	0.005	0.26
Amplifications				
Rab and other proteins involved in intracellular traffic	3	14	0.009	1
RNA binding genes	12	173	0.01	1

^a^By hypergeometric test without adjustment for testing of multiple gene sets

^b^Adjusted for multiple-testing by the Benjamini-Hochberg method

### Evidence of population-specific adaptation

**Fig. 3:**
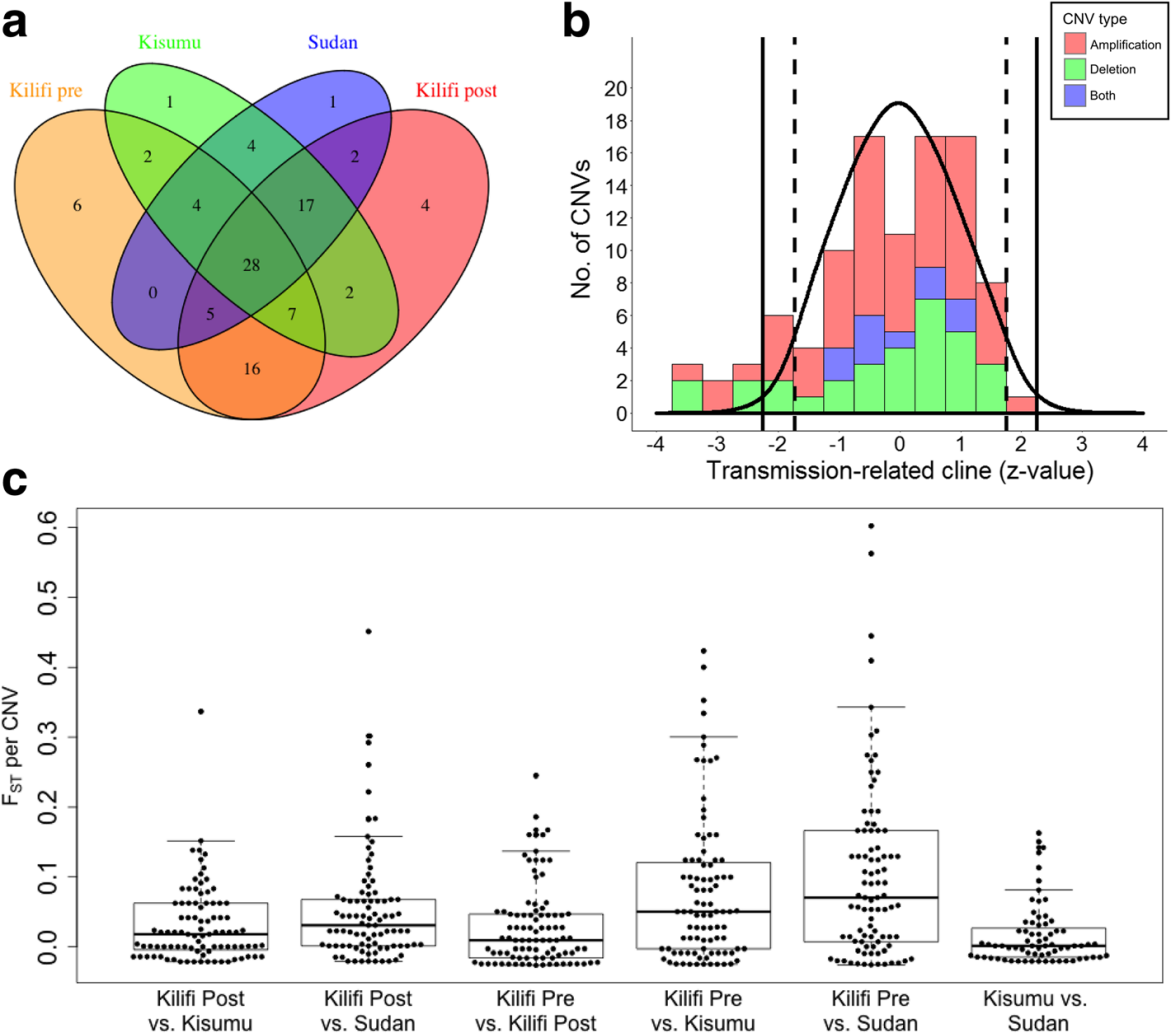
Population differentiation of CNVs. **a** Overlap of CNVs between populations. **b** Distribution of transmission intensity-related frequency clines (z-score) among the CNVs (filled bars, red for amplifications, green for deletions) vs. the expected distribution based on permuted data (black line). Vertical solid lines indicate the upper and lower 2.5% probability thresholds of the latter. Vertical dashed lines indicate the equivalent thresholds after Benjamini-Hochberg adjustment for multiple testing. **c** Distributions of population pairwise F_ST_ estimates for CNVs (dots, individual CNVs; horizontal line, median number; top and bottom boundaries, 75th and 25th percentiles, whiskers, minimum and maximum)

Many CNVs (28 of 99 when defining amp-dels as two separate CNVs) were present in all four populations and a few (12/99) were exclusive to single populations (Fig. 3a). Mean F_ST_ values ranged between 0.02 and 0.11 across the 6 pairwise population comparisons (Fig. 3c) which are typical values of background population differentiation in *P. falciparum* based on SNPs [33]. However, nine CNVs (9%) had pairwise F_ST_ values greater than the arbitrary significance threshold of 0.3, equivalent to the top 3% of all population pairwise values (Fig. 3c). These were thus declared as potential targets of population-specific selection (Table 3).

**Table 3:** CNVs showing evidence of population-specific adaptation. Only those CNVs that either had F_ST_ greater than 0.30 or which showed significant transmission intensity-related clines in population frequency are shown

CNV name	CNV type	F_ST_^a^	Cline^b^	Annotations^c^
cnv5_101	Amplification	0.64	- ***	6-cysteine protein (P38) SET domain protein, putative (SET9)
cnv12_413	Amplification	0.64	-*	
cnv9_242	Amplification	0.44		P1 nuclease, putative
cnv13_478	Deletion	0.46	- †	E1-E2 ATPase, putative
cnv9_254	Deletion	0.41		protein phosphatase-beta thioredoxin-like protein 2 (TLP2) zinc binding protein (Yippee), putative histone deacetylase 1 (HDAC1)
cnv3_051	Amplification	0.34		circumsporozoite- and TRAP-related protein (CTRP)
cnv3_036	Amplification	0.32	-†	Plasmodium exported protein (hyp1), unknown function (GEXP21)
				Plasmodium exported protein, unknown function
				Plasmodium exported protein, unknown function
cnv6_129	Deletion	0.30		ubiquitin-conjugating enzyme E2, putative polypyrimidine tract binding protein, putative
cnv4_078	Amplification	0.30	-†	
cnv11_354	Deletion	0.27		Plasmodium exported protein (PHISTc), unknown function (GEXP12)
				Plasmodium exported protein (hyp11), unknown function
				Plasmodium exported protein, unknown function
cnv11_355	Deletion	0.18	-***	antigen 332, DBL-like protein (Pf332)
cnv9_269	Deletion	0.27	-***	gametocyte development protein 1 (GDV1)
				Plasmodium exported protein, unknown function (GEXP22)
				gametocytogenesis-implicated protein (GIG)
				Plasmodium exported protein, unknown function
				cytoadherence linked asexual protein 9 (CLAG9)
				ring-exported protein 1 (REX1)
				ring-exported protein 2 (REX2)
				early transcribed membrane protein (ETRAMP9)
				ring-exported protein 4 (REX4)
				virulence-associated protein 1 (VAP1)
				Plasmodium exported protein (PHISTc), unknown function
				(GEXP05)
				lysophospholipase, putative
				Plasmodium exported protein (PHISTc), unknown function
				Plasmodium exported protein (PHISTb), unknown function
				Plasmodium exported protein, unknown function
				lysophospholipase, putative
cnv1_007	Amplification	0.26	-**	tubulin-specific chaperone a, putative N-acetyltransferase, putative
cnv14_549	Amplification	0.21	-***	thioredoxin peroxidase 1 (Trx-Px1) copper transporter
cnv11_355	Deletion	0.18	- ***	antigen 332-DBL-like protein (Pf332)
cnv2_023	Deletion	0.14	-*	conserved Plasmodium membrane protein, unknown function
cnv12_368	Deletion	0.07	-*	glycerol-3-phosphate dehydrogenase, putative

^a^Maximum of pairwise population F_ST_ values

^b^Significance of transmission intensity cline in CNV frequency after Benjamini-Hochberg correction for multiple testing. (†, *P* < 0.10;*, *P* < 0.05;**, *P* < 0.01;***, *P* < 0.001; +, positive association; −, negative association). Four further CNVs had clines with adjusted *P* < 0.10 but F_ST_ < 0.3 and so are not shown (three amplifications, cnv9_262, − †; cnv5_109, + †; cnv14_573, − †, and one deletion, cnv7_193, − †)

^c^Excluding genes with no known or putative function

A higher than expected number of CNVs showed significant transmission intensity-related clines in population frequency (*P* < 0.001 based on the distribution of the global test statistic from permuted data, Table 3, Fig. 3b). Nine CNVs (9%, 4 amplifications and 5 deletions) showed individual significance after adjustment for multiple testing (Benjamini-Hochberg adjusted *P* < 0.05). All of these CNVs decreased in frequency as transmission intensity increased (Table 3). One of these was the large deletion on the right arm of chromosome 9 (cnv9_269) that has previously been observed only in laboratory-adapted isolates [11, 12, 16, 18, 19, 34]. A further seven CNVs (6 with negative clines, 2 of which were deletions) had marginally significant frequency clines (adjusted *P* < 0.10). There was strong overlap in the CNVs displaying population differentiation by F_ST_ and those exhibiting transmission-related clines (Table 3).

These results strengthen the argument that CNVs play a role in local adaptation of *P. falciparum* to its natural environment. Our results suggest that a ‘landscape genomics’ approach applied to malaria parasite populations on a much larger scale than in this study might accelerate progress towards identification of genetic variants that enable the parasite to survive and thrive in its highly variable environment. Such an approach has been demonstrated as successful in identifying adaptive genes in humans affecting metabolic disease due to diet, altitude and heat [35, 36] and infectious diseases such as malaria [37].

### The chromosome 9 deletion

The most striking transmission-related frequency cline was in cnv9_269 which was found most often in Sudan (17 out of 52 isolates, 33%), but at low frequency in other populations (< 9%). Its reported absence from isolates taken directly from patients in previous studies has led to the interpretation that this deletion is an artefact of in vitro culture, assumed to arise from a replication advantage under these novel conditions, but strongly selected against in nature because it contains genes coding for several proteins essential for early gametocyte development [38, 39] and thus transmission. The deleted region also contains genes encoding proteins involved in cytoadherence [40], the in vivo process by which parasite-infected red cells adhere to vascular tissues and thereby protect parasite infected red cells from being circulated and destroyed by the spleen. Since cytoadherence is redundant in vitro*,* selection against this deletion would only be expected to occur in vivo, just as for gametocyte development genes. A replication advantage of this deletion in vitro might arise from the lower metabolic cost of DNA replication of a smaller genome. Alternatively, it may arise because the production of gametocytes imposes a cost on asexual replication [41]. These advantages would also be expected to apply in vivo.

We envisage two possible mechanisms for how this deletion could be maintained in natural populations despite its apparent cost to transmission and survival of host clearance mechanisms, and for why these may be more powerful in areas with low or strongly seasonal transmission intensities. First, the mutation may commonly arise de novo in new infections, rising to high within-host frequency due to a replication advantage, but ultimately being unable to transmit. Such ‘short-sighted, dead-end’ within-host evolution has been invoked to explain the high virulence of some pathogens [42]. In *Plasmodium*, such a scenario would be favoured when there are long mosquito-free periods between malaria transmission seasons, as occur in the eastern Sudan population examined here, because there is no transmission cost to counteract the short-term selection for rapid replication.

An alternative explanation is that genomes with inherent instability at the chromosome 9 locus are maintained in natural parasite populations through ‘bet hedging’. Under this scenario, within a host, a subset of asexual lineages deriving from the same parasite clone may carry the deletion while simultaneously maintaining intact lineages that are capable of transmitting. A bet-hedging strategy would be selectively favoured when there is no competition from co-infecting genotypes for uptake by the mosquito. It would also require that kin selection was at play, as appears to be the case for sex ratio adjustment by *Plasmodium* in response to the presence of unrelated genotypes [43].

Both these explanations are consistent with the high frequency of cnv9_269 in the highly seasonal setting of Sudan observed here. This observation also accords with the finding in two populations in west Africa of extreme F_ST_ values for five SNPs within and adjacent to the first gene in cnv9_269 (*gdv1*, encoding gametocyte development protein1, PF3D7_0935400). In this case, the minor allele was found more often in the population with strong malaria seasonality than in the population with year-round transmission [44], consistent with this study. Thus there is mounting evidence that this locus is the target of selection in highly seasonal and low transmission environments.

It seems likely that the cnv9_269 deletion has escaped detection in field isolates until now because for most detection methods, including CGH, its presence would be masked by non-deleted genomes in the parasite population in the blood. This masking effect would be strongest in high transmission areas where most infections are multi-clonal, and thus may have contributed to the observed negative frequency cline in cnv9_269 and other deletions found in this study.

### Associations between CNVs

**Fig. 4:**
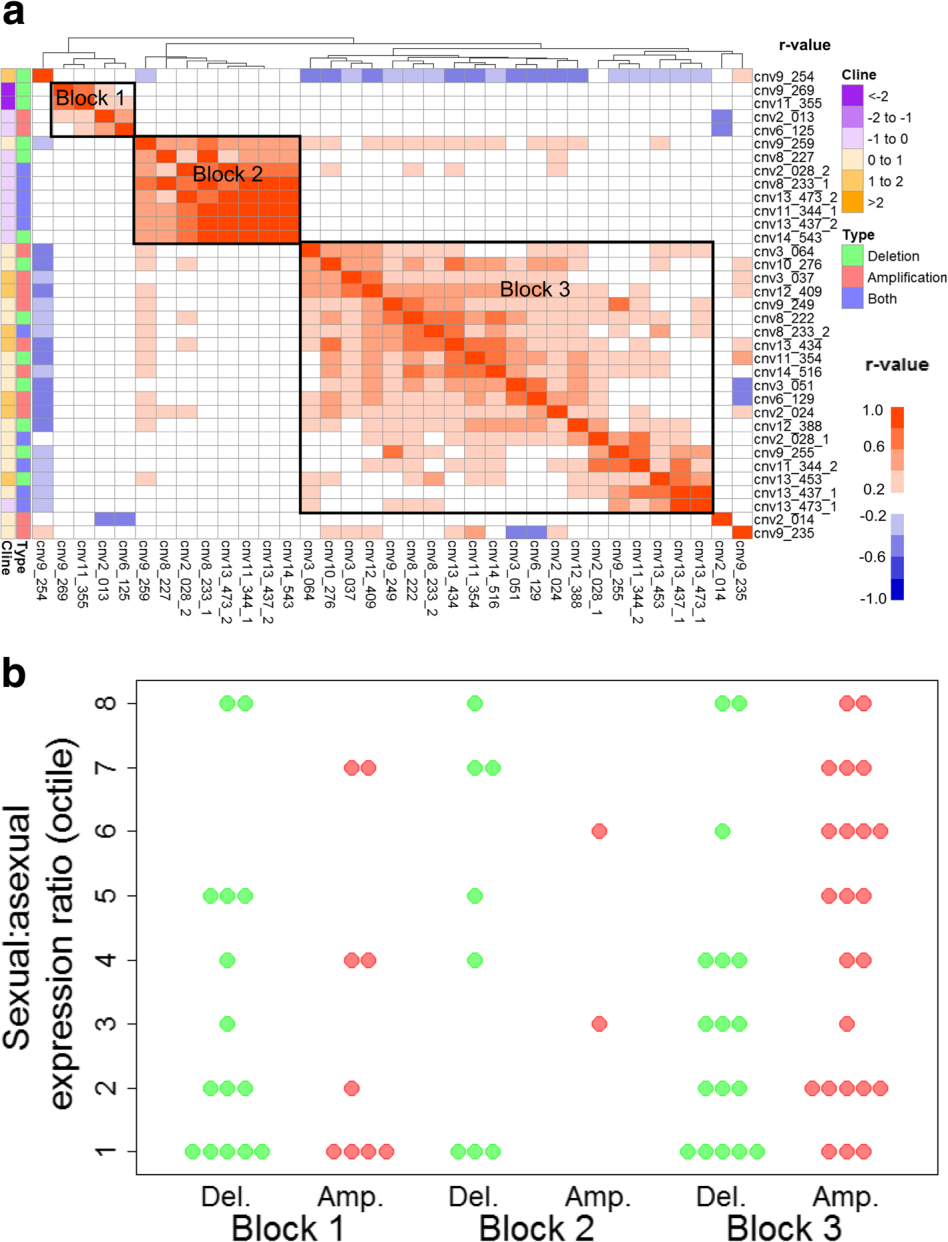
Associations between CNVs and sexual stage function. **a** pairwise linkage disequilibrium between CNV alleles. Heatmap colours indicate the strength and direction of the correlation between isolates across populations (r-value, white indicates the same CNV). Colour bars on left indicate the type of CNV and the strength and direction of its transmission intensity related frequency clines. CNVs were clustered (top dendrogram) by similarity in correlation profiles. CNVs with low linkage disequilibria are excluded. **b** Ratio of sexual to asexual stage expression (y-axis) for individual inside the genes in (**a**) grouped by linkage disequilibrium block and CNV type (Amp., amplification; Del., deletion)

Linkage disequilibrium analyses revealed three distinct sets of CNVs (Blocks 1 to 3) with strong population-level associations between them (0.6 < *r* < − 0.4) (Fig. 4a). The largest block (Block 3) contained approximately equal numbers of amplification and deletion CNVs which, respectively, typically contained genes with high and low sexual stage expression levels (Fig. 4b), and thus are denoted as ‘sexual stage CNVs’ here. There was a striking negative correlation between Block 3 CNVs and a deletion CNV that was not a member of any block, cnv9_254. The latter contains a gene encoding histone deacetylase 1 (HDAC1) which has been strongly implicated as the provider of epigenetic silencing that underpins the transcriptional programme of the intraerythrocytic 48 h asexual replication cycle and which appears to be switched off upon conversion to gametocytes [45]. Block 3 CNVs further showed a strong positive association with another deletion on chromosome 9, cnv9_259, which contains a gene encoding a component of cytochrome oxidase, an enzyme used in the energy-generating electron transport chain in the mitochondrion. Malaria parasites increase their dependence on mitochondrial activity upon conversion to gametocytes [46, 47]. Combined, the data suggest that cnv9_254 and cnv9_259 deletions, in conjunction with Block 3 CNVs, are involved in up-regulation of sexual stage activities.

This contrasts with Block 1 CNVs which associated with loss of sexual function and perhaps gain in asexual function. Block 1 contains cnv9_269, the chromosome 9 deletion causing loss of gametocyte production discussed above, and another deletion, cnv11_355, which, as for cnv9_269, contains a gene involved in export of proteins to the red cell surface, Pf332 [48]. Block 1 also includes a CNV on chromosome 2, cnv2_013, which, like cnv9_269, is frequently found in laboratory isolates adapted to in vitro culture. However, this CNV was amplified in field parasites, whereas in vitro, only deleted forms are found. cnv2_013 contains genes encoding KAHRP and PTP1, both of which are involved in export to the red cell surface and cytoadherence in asexual blood stage parasites [49, 50], and LSAP2, which is associated with liver stage infection [51]. Other genes contained within Block 1 amplification CNVs included the liver stage merozoite protein, PALM, the eukaryotic translation elongation factor EF-G, and geranylgeranyltransferase, all of which are highly expressed during the asexual blood or liver stages. Thus it appears that Block 1 amplifications are associated with functions relating to in vivo asexual replication and survival*,* including cytoadherence, while Block 1 deletions are associated with loss of sexual function, though also some components of cytoadherence. Both of the amplification CNVs in Block 1 (cnv2_013 and cnv6_125) showed a strong negative correlation with the CNV directly adjacent to cnv2_013, namely, cnv2_014. cnv2_014, also an amplification, contains two genes which are both abundantly expressed in gametocytes and ookinetes [28]. One of these genes encodes a protein found in the parasite surface membrane (ETRAMP2) which is expressed in mosquito stages, including sporozoites [52], and for which other family members [53], but not this [54], are essential for liver stage development in the rodent malaria parasite, *P. berghei*. This apparent antagonism between cnv2_014 and Block 1 amplifications bolsters support for our proposition that Block 1 CNVs create loss of sexual function and concomitant gain in asexual function.

An unexpectedly high proportion of amp-del CNVs were among those showing strong population-level associations (10 of all 10 amp-dels vs. 13 of 31 deletions and 12 of 58 amplifications, *P* < 0.001 by Fisher’s Exact test). The amp-dels fell mainly within Blocks 2 and 3, and clustered with amplification CNVs. We propose that amp-del CNVs alter their copy number negatively or positively to suit the prevailing functional needs of asexual vs. sexual parasites, as driven by CNVs in Block 1 and Block 3.

We interpret the associations between sets of CNVs in population prevalence as the outcome of co-selection on reproductive vs. replicative investment which differs according to local transmission intensity. In a parallel study on gene expression levels, we have shown that parasites in low transmission areas invest more in reproduction and less in asexual replication than parasites from high transmission areas [55]. We cannot say which environmental factors wield the strongest selective forces, but the most likely candidates are average infection intensities (and hence in-host competition levels), levels of host population immunity, host genetics, drug treatment and transmission opportunities, all of which vary widely between geographical areas and lead to different benefit cost-ratios of reproduction and replication [4]. Alternatively, it is possible that associations between CNV subsets are generated by an over-arching mechanism that coordinates the spontaneous induction of sets of functionally related CNVs. This seems less likely, but not impossible since some strongly correlated CNVs lie adjacent to each other on the chromosome. In particular, CNVs on chromosome 9 were influential and antagonistic, perhaps suggesting that they arise through remodeling of this chromosome at the time of switching from asexual replication to sexual reproduction.

## Conclusions

The results of this study show that gene copy number variation is common in natural populations of *P. falciparum* parasites, consistent with previous studies. They also provide, for the first time, evidence that CNVs in *Plasmodium* provide adaptive value in the face of natural selection pressures in the parasite’s field environment. This evidence is based on observations of more than expected CNVs which display transmission intensity-related population differentiation, of strong population-level associations between CNVs, and that these CNVs contain genes which directly affect short-term in-host fitness (replication) and longer term between-host fitness (reproduction).

We interpret population differences in frequencies of CNVs as the product of three components, namely, the inherent trade-off between asexual replication and reproduction in the parasite’s life cycle; the conflict between short-term (in-host) and long-term (between-host) fitness; and the different benefit-cost ratios of these fitness components in different transmission environments. For example, in the case of cnv9_269, the sacrifice of gametocyte production incurs little cost in environments with few mosquitoes, thus allowing short-term selection that favours asexual replication to dominate over longer term selection for transmission to new hosts. Our finding that CNVs cluster according to reproductive vs. replicative functions, and that there are antagonistic associations among deletions that are expected to nullify these functions, suggests that the short-term selection argument generalizes to other CNVs too, generating co-selected suites of CNVs specialized for these two highly differentiated life stages.

It is difficult to explain how CNVs that cause loss of function are maintained in the general population in the field. We have proposed that this could be achieved through maintenance of genomic fragility at CNV loci that would allow ‘bet-hedging’ within an infection. Under this strategy, the parasite would divide its asexually replicating lineages into mutant and non-mutant types, thereby allowing maintenance of both asexual replication and reproduction and thus carryover to the next generation. Splitting of function in this way is akin to somatic differentiation of tissues in multi-cellular organisms which allows a balance between growth and reproduction to be achieved in order to maximize lifetime fitness. However, in *Plasmodium,* it is fitness of the individual parasite, not the population of parasites within the infection, which is rewarded. Although kin selection has been proposed to play a role in the evolution of life history traits in *Plasmodium* [56], there are few empirical studies to test this. Moreover, it is clear that competition between different genotypes occupying the same host is a strong determinant of the fitness of individual parasite genotypes [57]. This leads us to conclude that CNVs that abrogate sexual function are likely to be the outcome of short-term selection only in the limited situation where infections are clonal and when opportunities for transmission are extremely low. By contrast, we interpret the finding of CNVs associated with enhanced reproductive function as the outcome of selection for between-host transmission when there are regular transmission opportunities and the benefits of switching to reproduction outweigh the costs of reduced asexual replication [41, 58].

This study has some limitations. First, although high stringency was applied in defining CNVs (this by applying high thresholds for significance, filtering out probes targeting highly polymorphic genes, probes with known SNPs within the probe sequences, poorly hybridizing probes and low frequency CNVs), it cannot be ruled out that unaccounted for DNA sequence variation in the field isolates caused poor probe hybridization thereby leading to false CNVs. Studies of probe hybridization as a function of number of base pair differences between probe and target suggest that 7 out of the 70 bases would have to be different in order to cause non-hybridisation [59]. Second, CNVs in the reference parasite (P4) genome may have led to an over- or underestimation of CNV prevalence. To account for this, we reported CNV frequencies with respect to the allele with the minor frequency. Third, microarray data provide low resolution of CNV breakpoints, leading to potentially incorrect start and endpoints of a CNV and hence lower accuracy of detection. Finally, choice of reference material, statistical methods, power, significance thresholds, platforms and technologies all differ widely between studies, thus eroding comparability across studies, especially for CNVs not validated through other methods. Although the chromosome 9 deletion is well validated by a variety of detection technologies, including the array used here [18], it is important that the novel finding in this study of its presence in field populations is tested through independent investigations.

Overall, this study shows that CNVs contribute substantially to levels of standing genetic variation in *P. falciparum* in natural populations and provides multiple lines of evidence that some of these CNVs are adaptive in the face of geographic and temporal variation in the parasite’s transmission environment. Further investigation of CNV genes in relation to gene expression levels, of their broader phenotypes, and of the specific selection pressures that mould their population frequencies will provide new leads on molecular mechanisms that allow malaria parasites to survive and adapt, ultimately leading to new ways to control malaria.

## Methods

### Sample population

Parasites were obtained by venesection of < 3 ml of blood from patients diagnosed with *P. falciparum* malaria by microscopy that attended healthcare facilities with symptoms. They were recruited from three areas in Eastern Africa, namely, eastern Sudan (Gedaref, Kassab, Medani, recruited in October 2007), western Kenya (Kisumu, recruited in April–May 2008) and coastal Kenya (Kilifi, recruited in April–May 2010). These areas have maintained low, high and moderate malaria transmission intensities, respectively, over a long period [55]. In Kilifi, archived parasite samples collected from patients recruited from the hospital 15 years previously (1994 to 1996), when transmission intensity was much higher than in 2010 [60], were also analysed: this gave a further contrast of medium-high (“Kilifi-pre”) vs. medium-low (“Kilifi-post”) transmission populations.

### Sample processing

After centrifugation, the plasma and buffy coat were removed in order to minimize contaminating human host DNA. For samples from Kilifi, 30–200 μl of parasite-infected red blood cells (iRBCs) were stored frozen then thawed on ice and saponified in order to remove intact parasites from RBCs. From this lysate, genomic DNA was extracted using the phenol chloroform method. For samples from Sudan and Kisumu, DNA was extracted from 100 μl of iRBCs, which had been stored frozen, using the automated ABI PRISM 6100 Nucleic Acid PrepStation (Applied Biosystems). The number of parasite clones in an isolate was determined by genotyping *P. falciparum* merozoite surface antigen 2 (*msp2*) gene [61].

### Comparative genomic hybridization

The microarray used for this study consisted of 70mer oligonucleotides (probes) spotted on a glass slide [59]. The probes on the array were designed using the available complete *P. falciparum* genome sequence of 3D7 parasite line [62] targeting conserved regions of approximately 5400 genes with an average of two probes per gene [59]. This array has been previously validated for detection of CNVs [18, 63]. Comparative genomic hybridization (CGH) was performed on 183 samples using as a reference the laboratory culture-adapted line, P4, that originated from a malaria patient at the Kilifi District Hospital [18]. To increase the amount of DNA available for hybridization to the array, whole genome amplification using random nonamers was performed [64]. Samples were randomized across population groups during amplification and hybridization experiments to avoid batch-of-processing bias. Cyanine fluorescent dyes (Cy3 for reference DNA and Cy5 for test DNA) were used for DNA labelling using the Klenow fragment. PCR amplification was terminated after 19 cycles (during the linear phase) in order to preserve the starting values of relative DNA abundance per gene. Competitive hybridization of each of the test samples against the reference was performed on a MAUI 12-bay hybridization station (BioMicro Systems). Microarray slides were scanned and analysed using GenePix 4000B microarray scanner and its software (version 4.0).

### Pre-processing of microarray data

Analysis of the microarray data was performed using the *limma* package in R [65]. First, poor quality spots (less than 6 pixels, or with size that greatly differed from that in the GAL file) were filtered out of the data. Second, data were normalised for spot intensity within arrays using the ‘normexp’ [66] and ‘robustspline’ [67] methods. Third, data were normalized for between-array variation using the “quantile” method. Data from genes encoding the variant antigen gene families of *var.*, *rifin* and *stevor*, and other multi-copy or highly variable genes, ribosomal RNAs and transfer RNAs were excluded from further analyses.

### Detection of gene copy number variation using R-GADA

Genomic regions that varied in copy number were identified using the Genome Alteration Detection Analysis (GADA) program in R [68]. The GADA method identifies contiguous segments in the genome in which log_2_ intensity differs from that of flanking genes. ‘Significant’ segments were declared based on a t-statistic calculated from the mean and variance of all the segments (‘T’) after applying segmentation analysis with segment length being controlled by the parameter a_α_. Here, we used the recommended thresholds for high sensitivity but also high false discovery rate of *T* = 3.5 and a_α_ [69]. To reduce false discovery rates, we filtered out segments with less than two microarray probes within the segment and an absolute amplitude of log_2_ ratio of < 1 (< 2-fold change in gene copy number). Since GADA CNV breakpoint predictions are not precise, and breakpoints also can vary between samples for biological reasons, locations of the start and end points of segments varied between samples. Therefore, segments with overlapping locations across samples and of similar types (i.e., amplification vs. deletion) were merged into a single CNV: this further protected against false discoveries. Finally, CNVs found in less than 4 out of 183 isolates were excluded from the final list of CNVs used in subsequent analyses.

### Systematic effects of experimental, host and gene factors

Population prevalence of each CNV – where population is defined as number of hosts as compared with number of distinct parasite genomes - was analysed as a binary variable (present vs. absent) for the systematic effects of population, multiplicity of infection (MOI), haemoglobin, age of participant, parasitaemia, experimental batch and parasite isolate using mixed effects logistic regression models in the *lme4* package in R [70]. All effects were fitted as fixed-level factors with the exception of batch and isolate which were fitted as random effects, the latter to allow for repeated measures on the same parasite material. The same model was fitted to data from all CNVs simultaneously but with further inclusion of CNV identifier as a random effect: this was to test for generalized bias from the above factors in overall CNV detectability while accounting for repeated measures on the same CNV. Significance of fixed effects was assessed by analysis-of-variance F-tests.

Prevalence of CNVs in the genome was analysed for the systematic effects of the following gene properties: SNP density (obtained from PlasmoDB), ratio of expression during the sexual vs. asexual stages of the life cycle (obtained from [71]), and the stage during the 48 h asexual replication cycle at which it was maximally expressed (based on data in [55]). A logistic regression model was fitted to the binary variable of whether the gene was a member of a CNV or not fitting fixed effects for fixed-level factors for the gene property traits above. Analyses were performed separately for CNVs that were deletions vs. amplifications: CNVs exhibiting both of these were ignored. Significance was assessed by analysis-of-deviance likelihood ratio tests. For all models, least-squares means were calculated for each level of the fixed effects using the *lsmeans* package in R [72].

### Functional enrichment

Enrichment for function among genes identified to be copy number variable was assessed by hypergeometric test for over-representation of CNVs among sets of functionally related genes, implemented by using the ‘phyper’ function in the *stats* package in R [73] and corrected for multiple testing using the Benjamini-Hochberg method [74]. Gene sets were constructed from the Malaria Parasite Metabolic Pathways database [75] and further categorized into higher level functional groupings as described in [55].

### Testing for evidence of population-level adaptation

To test for evidence of CNV-related population level adaptation in general, Weir and Cockerham F-statistics (F_ST_) for levels of between-to-within population variation in allele frequencies were calculated for each CNV using *hierfstat* as implemented in R [76]. CNVs with unusually high or low values, and thus potential targets of directional and diversifying selection, respectively, were identified by comparing them to the distribution of F_ST_ values for all CNVs. To determine whether these population differences were related to transmission intensity in the population, a fixed effects logistic regression model was fitted to prevalence data for each CNV with population fitted as a linear covariate representing the populations’ ranks in transmission intensity, i.e., 1 to 4 for Sudan, Kilifi-post, Kilifi-pre and Kisumu respectively. This model was fitted to data on all CNVs simultaneously, with CNV fitted as a fixed effect and the population covariate fitted within CNV. The standardized regression slopes (z-score) for each CNV, which represent the cline in CNV frequency across the transmission intensity gradient, were compared to a null distribution of slopes constructed by fitting the same model to 1000 permutations of the data in which population membership of each parasite isolate had been randomly reassigned. Unadjusted *P*-values for regression slopes were based on t-tests using the error variance from all CNVs combined. To allow for multiple testing, P-values were adjusted to reflect a false discovery rate using the Benjamini-Hochberg method [74].To test whether the observed distribution of slopes for all the CNVs differed from that expected by chance, a global test statistic, namely, the sum of the absolute z-scores, was computed for the observed data and compared to the distribution of this statistic from the permuted data. CNVs with significant clines (adjusted *P* < 0.05) or F_ST_ values > 0.3 for at least one of their pairwise population values were defined as ‘adaptive’.

### Linkage disequilibrium

To test for population level associations between CNVs, linkage disequilibria between all pairwise combinations of CNVs were calculated using the *pegas* package in R [77]. Pearson correlations were computed between frequencies of the CNVs’ minor alleles (r-values). CNVs with both amplification and deletion alleles were treated separately. Results were visualized as a heatmap using the *pheatmap* package in R.

## Additional file

Additional file 1: CNVs found in this study and their characteristics. Names of CNVs, their type (amplification or deletion), the genes contained within them and previous reports in the literature. (PDF 175 kb)

## Abbreviation

CNV: Copy number variant
