# Supplementary material for: Gene copy number variation in natural populations of Plasmodium falciparum in Eastern Africa
Source: BMC Genomics. 2018 May 21;19:372. doi: 10.1186/s12864-018-4689-7 (PMC5963192; doi:10.1186/s12864-018-4689-7)
Supplement: Supplementary file 1 — CNVs found in this study and their characteristics. Names of CNVs, their type (amplification or deletion), the genes contained within them and previous reports in the literature. (PDF 175 kb) [file 12864_2018_4689_MOESM1_ESM.pdf]

CNV name	Type	Chromo some	Gene ID	Annotation	Previous reports
**cnv1_005**	Amplification	1	PF3D7_0108500	conserved Plasmodium protein, unknown function	[2]
			PF3D7_0108600	conserved Plasmodium protein, unknown function	[1,4,7,8-11]
cnv1_007	Amplification	1	PF3D7_0109400	tubulin-specific chaperone a, putative	
			PF3D7_0109500	N-acetyltransferase, putative	
**cnv2_013**	Amplification	2	PF3D7_0202000	knob-associated histidine-rich protein (KAHRP)	[1,4,7,8-11]
			PF3D7_0202100	liver stage associated protein 2 (LSAP2)	[1,2,6]
			PF3D7_0202200	EMP1-trafficking protein (PTP1)	[3,6,8]
cnv2_014	Amplification	2	PF3D7_0202500	early transcribed membrane protein 2 (ETRAMP2)	
			PF3D7_0202600.1	conserved Plasmodium protein, unknown function	
cnv2_016	Amplification	2	PF3D7_0203100	protein kinase, putative	
cnv2_023	Deletion	2	PF3D7_0212400	conserved Plasmodium membrane protein, unknown function	
cnv2_024	Amplification	2	PF3D7_0213600	conserved Plasmodium protein, unknown function	
cnv2_028_1	Amplification	2	PF3D7_0215700	DNA-directed RNA polymerase II subunit RPB2, putative (RPB2)	
cnv2_028_2	Deletion	2	PF3D7_0215700	DNA-directed RNA polymerase II subunit RPB2, putative (RPB2)	
cnv3_036	Amplification	3	PF3D7_0301600	Plasmodium exported protein (hyp1), unknown function (GEXP21)	
			PF3D7_0301700	Plasmodium exported protein, unknown function	
			PF3D7_0301800	Plasmodium exported protein, unknown function	
cnv3_309	Deletion	3	PF3D7_0302500	cytoadherence linked asexual protein 3.1 (CLAG3.1)	
cnv3_037	Amplification	3	PF3D7_0302600	ABC transporter B family member 4, putative (ABCB4)	
			PF3D7_0302700	CDGSH iron-sulfur domain-containing protein, putative	
			PF3D7_0302800	conserved Plasmodium protein, unknown function	
			PF3D7_0302900	exportin-1, putative	
**cnv3_043**	Deletion	3	PF3D7_0309300	N2227-like protein, putative	[6]
			PF3D7_0309500	asparagine synthetase, putative	[3]
			PF3D7_0309600	60S acidic ribosomal protein P2 (PfP2)	
cnv3_051	Deletion	3	PF3D7_0315200	circumsporozoite- and TRAP-related protein (CTRP)	
cnv3_064	Amplification	3	PF3D7_0322700	conserved Plasmodium protein, unknown function	
cnv4_073	Amplification	4	PF3D7_0407000	conserved Plasmodium protein, unknown function	
			PF3D7_0407100	methyltransferase, putative	
			PF3D7_0407200	peptidyl-tRNA hydrolase 2, putative (PTH2)	
cnv4_076	Amplification	4	PF3D7_0409500	conserved Plasmodium protein, unknown function	
			PF3D7_0409500	conserved Plasmodium protein, unknown function	

CNV name	Type	Chromo some	Gene ID	Annotation	Previous reports
			PF3D7_0409600	replication protein A1, large subunit (RPA1)	
			PF3D7_0409700	peptide chain release factor 2, putative	
cnv4_078	Amplification	4	PF3D7_0411700	conserved Plasmodium protein, unknown function	
**cnv4_091**	Amplification	4	PF3D7_0423400	asparagine-rich protein (AARP)	[10]
			PF3D7_0423500	glideosome associated protein with multiple membrane spans 2 (GAPM	[10]
**cnv4_092**	Amplification	4	PF3D7_0424400	surface-associated interspersed protein 4.2 (SURFIN 4.2) (SURF4.2)	[4,10]
cnv5_101	Amplification	5	PF3D7_0507900	conserved Plasmodium protein, unknown function	
			PF3D7_0508000	6-cysteine protein (P38)	
			PF3D7_0508100	SET domain protein, putative (SET9)	
cnv5_106	Amplification	5	PF3D7_0512600	ras-related protein Rab-1B (RAB1b)	
			PF3D7_0512700	orotate phosphoribosyltransferase (OPRT)	
cnv5_108	Amplification	5	PF3D7_0514300	aspartate--tRNA ligase, putative	
			PF3D7_0514500	conserved Plasmodium membrane protein, unknown function	
			PF3D7_0514600	ribose 5-phosphate epimerase, putative	
cnv5_109	Amplification	5	PF3D7_0515000	pre-mRNA-splicing factor CWC2, putative (CWC2)	
			PF3D7_0515100	rhomboid protease ROM9 (ROM9)	
			PF3D7_0515200	conserved Plasmodium protein, unknown function	
			PF3D7_0515300	phosphatidylinositol 3-kinase (PI3K)	
**cnv5_122**	Deletion	5	PF3D7_0529000	conserved Plasmodium protein, unknown function	[4]
			PF3D7_0529100	conserved Plasmodium protein, unknown function	[6]
			PF3D7_0529200	sugar transporter, putative	
cnv6_125	Amplification	6	PF3D7_0602000	conserved Plasmodium protein, unknown function	
			PF3D7_0602100	ATP-dependent RNA helicase, putative	
			PF3D7_0602200	MYND finger protein, putative	
			PF3D7_0602300	liver merozoite formation protein, putative (PALM)	
			PF3D7_0602400	elongation factor G (EF-G)	
			PF3D7_0602500	geranylgeranyltransferase, putative	
cnv6_127	Amplification	6	PF3D7_0604800	RAP protein, putative	
			PF3D7_0604900	conserved Plasmodium protein, unknown function	
			PF3D7_0605000	50S ribosomal protein L24, putative	
			PF3D7_0605100	RNA-binding protein, putative	
cnv6_129	Amplification	6	PF3D7_0606200	ubiquitin-conjugating enzyme E2, putative	

CNV name	Type	Chromo some	Gene ID	Annotation	Previous reports
			PF3D7_0606300	conserved Plasmodium protein, unknown function	
			PF3D7_0606400	conserved Plasmodium protein, unknown function	
			PF3D7_0606500	polypyrimidine tract binding protein, putative	
**cnv7_169**	Amplification	7	PF3D7_0710100	conserved Plasmodium protein, unknown function	[4,6,10]
			PF3D7_0710200	conserved Plasmodium protein, unknown function	
**cnv7_181**	Amplification	7	PF3D7_0721000	conserved Plasmodium membrane protein, unknown function	[10]
			PF3D7_0721100	conserved Plasmodium protein, unknown function	
cnv7_185	Amplification	7	PF3D7_0724100	conserved Plasmodium protein, unknown function	
			PF3D7_0724200	type 2A phosphatase-associated protein 42, putative (TAP42)	
cnv7_193	Deletion	7	PF3D7_0730500	conserved Plasmodium protein, unknown function	
**cnv8_201**	Deletion	8	PF3D7_0804700	conserved Plasmodium protein, unknown function	[6]
			PF3D7_0804800	peptidyl-prolyl cis-trans isomerase (CYP24)	
			PF3D7_0804900	GTPase-activating protein, putative	
			PF3D7_0805000	alpha/beta hydrolase, putative	
			PF3D7_0805100	conserved Plasmodium protein, unknown function	
**cnv8_215**	**Amplification**	**8**	**PF3D7_0818000**	**conserved protein, unknown function**	[6]
cnv8_222	Deletion	8	PF3D7_0822600	protein transport protein SEC23 (SEC23)	
cnv8_227	Deletion	8	PF3D7_0825900	conserved Plasmodium protein, unknown function	
			PF3D7_0826000	conserved Plasmodium protein, unknown function	
cnv8_233_1	Deletion	8	PF3D7_0829500	conserved Plasmodium protein, unknown function	
cnv8_233_2	Amplification	8	PF3D7_0829500	conserved Plasmodium protein, unknown function	
cnv9_235	Amplification	9	PF3D7_0902900	conserved Plasmodium protein, unknown function	
			PF3D7_0903000	conserved protein, unknown function	
			PF3D7_0903100	protein RER1, putative (RER1)	
cnv9_242	Amplification	9	PF3D7_0908000	P1 nuclease, putative	
			PF3D7_0908100	conserved Plasmodium membrane protein, unknown function	
cnv9_249	Amplification	9	PF3D7_0915200	ribonuclease H2 subunit C, putative	
			PF3D7_0915300	conserved Plasmodium protein, unknown function	
cnv9_251	Amplification	9	PF3D7_0920900	U4/U6 snRNA-associated-splicing factor, putative (PRP24)	
			PF3D7_0921000.1	ubiquitin-conjugating enzyme, putative	
			PF3D7_0921100	conserved Plasmodium protein, unknown function	
cnv9_254	Deletion	9	PF3D7_0925400	protein phosphatase-beta	

CNV name	Type	Chromo some	Gene ID	Annotation	Previous reports
			PF3D7_0925500	thioredoxin-like protein 2 (TLP2)	
			PF3D7_0925600	zinc binding protein (Yippee), putative	
			PF3D7_0925700	histone deacetylase 1 (HDAC1)	
cnv9_255	Deletion	9	PF3D7_0926400	monocarboxylate transporter, putative	
cnv9_259	Deletion	9	PF3D7_0928000.1	cytochrome c oxidase subunit 6B, putative (COX6B)	
cnv9_262	Amplification	9	PF3D7_0928900	guanylate kinase (GK)	
			PF3D7_0929000	transcription initiation factor TFIID subunit 7, putative (TAF7)	
			PF3D7_0929100	conserved Plasmodium protein, unknown function	
			PF3D7_0929200	RNA-binding protein, putative	
			PF3D7_0929300	conserved Plasmodium protein, unknown function	
cnv9_268	Amplification	9	PF3D7_0934300	conserved Plasmodium protein, unknown function	
			PF3D7_0934400	transcription factor with AP2 domain(s), putative (ApiAP2)	
**cnv9_269**	Deletion	9	PF3D7_0935400	gametocyte development protein 1 (GDV1)	[1,5,6,9,10]
			PF3D7_0935500	Plasmodium exported protein, unknown function (GEXP22)	[1,4,6,9,10]
			PF3D7_0935600	gametocytogenesis-implicated protein (GIG)	[1,2,4,6,9,10]
			PF3D7_0935700	Plasmodium exported protein, unknown function	[1,2,4,6,9,10]
			PF3D7_0935800	cytoadherence linked asexual protein 9 (CLAG9)	[1,2,4,6,9,10]
			PF3D7_0935900	ring-exported protein 1 (REX1)	[1,4,6,9,10]
			PF3D7_0936000	ring-exported protein 2 (REX2)	[1,2,4,6,9,10]
			PF3D7_0936100	early transcribed membrane protein (ETRAMP9)	[1,2,4,6,9,10]
			PF3D7_0936400	ring-exported protein 4 (REX4)	[1,2,4,6,9,10]
			PF3D7_0936500	virulence-associated protein 1 (VAP1)	[1,2,4,6,9,10]
			PF3D7_0936700	lysophospholipase, putative	[1,4,5,9,10]
			PF3D7_0936800	Plasmodium exported protein (PHISTc), unknown function	[1,2,4,6,9,10]
			PF3D7_0937000	Plasmodium exported protein (PHISTb), unknown function	[1,2,4,6,9,10]
			PF3D7_0937100	Plasmodium exported protein, unknown function	[1,2,4,6,9,10]
cnv10_270	Deletion	10	PF3D7_1001300	Plasmodium exported protein (PHISTa), unknown function	
			PF3D7_1001400	alpha/beta hydrolase, putative	
cnv10_276	Deletion	10	PF3D7_1008700	tubulin beta chain	
			PF3D7_1008800	nucleolar protein 5, putative (NOP5)	
			PF3D7_1008900	adenylate kinase (AK1)	
cnv10_279	Deletion	10	PF3D7_1012500	phosphoglucomutase, putative	

CNV name	Type	Chromo some	Gene ID	Annotation	Previous reports
cnv10_283	Deletion	10	PF3D7_1016500	Plasmodium exported protein (PHISTc), unknown function	
			PF3D7_1016700	Plasmodium exported protein (PHISTc), unknown function	
cnv11_304	Deletion	11	PF3D7_1107300	polyadenylate-binding protein-interacting protein 1, putative (PAIP1)	
			PF3D7_1107400	DNA repair protein RAD51 (RAD51)	
			PF3D7_1107500	prefoldin, putative	
cnv11_313	Deletion	11	PF3D7_1113800	conserved Plasmodium membrane protein, unknown function	
			PF3D7_1113900	mitogen-activated protein kinase 2 (MAPK2)	
cnv11_316	Deletion	11	PF3D7_1114800	glycerol-3-phosphate dehydrogenase, putative (G3PDH)	
			PF3D7_1114900	conserved Plasmodium protein, unknown function	
**cnv11_335**	Amplification	11	PF3D7_1128700	GPI-anchor transamidase (GPI8)	
			PF3D7_1128800	ribosome biogenesis regulatory protein, putative	
			PF3D7_1128900	conserved Plasmodium protein, unknown function	
			PF3D7_1129000	spermidine synthase (SpdSyn)	[6]
			PF3D7_1129100	parasitophorous vacuolar protein 1 (PV1)	
			PF3D7_1129200	26S proteasome regulatory subunit RPN7, putative (RPN7)	
**cnv11_344_1**	Deletion	11	PF3D7_1140500	myosin F, putative (MyoF)	[6]
			PF3D7_1140600	conserved Plasmodium protein, unknown function	
**cnv11_344_2**	Amplification	11	PF3D7_1140500	myosin F, putative (MyoF)	[6]
			PF3D7_1140600	conserved Plasmodium protein, unknown function	
cnv11_347	Amplification	11	PF3D7_1142600	60S ribosomal protein L35ae, putative	
			PF3D7_1142700	methyltransferase, putative	
			PF3D7_1142800	conserved Plasmodium protein, unknown function	
			PF3D7_1142900	conserved Plasmodium protein, unknown function	
			PF3D7_1143000	alpha/beta hydrolase, putative	
**cnv11_348**	Amplification	11	PF3D7_1143300	DNA-directed RNA polymerase I, putative	
			PF3D7_1143400	translation initiation factor eIF-1A, putative	
**cnv11_354**	Deletion	11	PF3D7_1148700	Plasmodium exported protein (PHISTc), unknown function (GEXP12)	[3,4,9,10]
			PF3D7_1148800	Plasmodium exported protein (hyp11), unknown function	[2,3,4,9]
			PF3D7_1148900	Plasmodium exported protein, unknown function	[3,4,10]
**cnv11_355**	Deletion	11	PF3D7_1149000	antigen 332, DBL-like protein (Pf332)	[3,4,10]
cnv12_359	Amplification	12	PF3D7_1201700	conserved Plasmodium membrane protein, unknown function	
			PF3D7_1201800	cytochrome c oxidase assembly protein COX19, putative (COX19)	

CNV name	Type	Chromo some	Gene ID	Annotation	Previous reports
			PF3D7_1201900	conserved protein, unknown function	
			PF3D7_1202000	ATP-dependent RNA helicase, putative	
cnv12_360	Amplification	12	PF3D7_1202100.1	SRAP domain-containing protein, putative	
			PF3D7_1202200	mitochondrial phosphate carrier protein	
cnv12_364	Amplification	12	PF3D7_1205400	conserved Plasmodium protein, unknown function	
			PF3D7_1205500	zinc finger protein, putative	
cnv12_367	Amplification	12	PF3D7_1207000	conserved Plasmodium protein, unknown function	
cnv12_368	Deletion	12	PF3D7_1207200	conserved Plasmodium protein, unknown function	
**cnv12_375**	Amplification	12	PF3D7_1212300	WD repeat-containing protein, putative	
			PF3D7_1212400	tetratricopeptide repeat family protein, putative	[6]
cnv12_377	Deletion	12	PF3D7_1214900	conserved Plasmodium membrane protein, unknown function	
			PF3D7_1215000	thioredoxin peroxidase 2 (Trx-Px2)	
cnv12_378	Amplification	12	PF3D7_1216200	glycerol-3-phosphate dehydrogenase, putative	
cnv12_379	Amplification	12	PF3D7_1217500	conserved Plasmodium protein, unknown function	
			PF3D7_1217600	anaphase promoting complex subunit 10, putative	
cnv12_388	Deletion	12	PF3D7_1229300	conserved Plasmodium protein, unknown function	
			PF3D7_1229400	macrophage migration inhibitory factor (MIF)	
			PF3D7_1229500	T-complex protein 1 subunit gamma (CCT3)	
cnv12_398	Deletion	12	PF3D7_1238900	protein kinase 2 (PK2)	
cnv12_405	Deletion	12	PF3D7_1243700	ubiquitin-conjugating enzyme E2, putative	
cnv12_408	Amplification	12	PF3D7_1244700	conserved Plasmodium protein, unknown function	
			PF3D7_1244800	cytoplasmic translation machinery associated protein, putative	
cnv12_409	Amplification	12	PF3D7_1245200	conserved Plasmodium membrane protein, unknown function	
cnv12_413	Amplification	12	PF3D7_1248600	conserved Plasmodium protein, unknown function	
cnv13_428	Amplification	13	PF3D7_1309000	conserved Plasmodium protein, unknown function	
			PF3D7_1309100	60S ribosomal protein L24, putative	
			PF3D7_1309200	protein phosphatase PPM6, putative (PPM6)	
			PF3D7_1309300	U4/U6 small nuclear ribonucleoprotein PRP3, putative (PRPF3)	
**cnv13_434**	Amplification	13	PF3D7_1312800	conserved Plasmodium protein, unknown function	
			PF3D7_1312900	eukaryotic translation initation factor 4 gamma (EIF4G)	
			PF3D7_1313000	ubiquitin-like protein nedd8 homologue, putative (Nedd8)	
			PF3D7_1313100	conserved Plasmodium protein, unknown function	[6]

CNV name	Type	Chromo some	Gene ID	Annotation	Previous reports
			PF3D7_1313200	methionyl-tRNA formyltransferase, putative (MTFMT)	
cnv13_437_1	Deletion	13	PF3D7_1315400	zinc finger (CCCH type) protein, putative	
cnv13_437_2	Amplification	13	PF3D7_1315400	zinc finger (CCCH type) protein, putative	
**cnv13_441**	Amplification	13	PF3D7_1316800	protein transport protein SEC20, putative (SEC20)	[6]
			PF3D7_1316900	conserved Plasmodium protein, unknown function	
			PF3D7_1317000	U4/U6.U5 tri-snRNP-associated protein 2, putative (USP39)	
cnv13_442	Amplification	13	PF3D7_1317300	conserved Plasmodium protein, unknown function	
			PF3D7_1317400	conserved Plasmodium protein, unknown function	
cnv13_447	Deletion	13	PF3D7_1322300	translation initiation factor EIF-2B subunit related	
cnv13_453	Deletion	13	PF3D7_1324900	L-lactate dehydrogenase (LDH)	
			PF3D7_1325000	U6 snRNA-associated Sm-like protein LSm6, putative (LSM6)	
cnv13_457	Amplification	13	PF3D7_1329200	conserved Plasmodium protein, unknown function	
			PF3D7_1329300	chromatin assembly factor 1 subunit, putative	
cnv13_469	Deletion	13	PF3D7_1340400	conserved Plasmodium protein, unknown function	
cnv13_473_1	Deletion	13	PF3D7_1344600	lipoyl synthase (LipA)	
cnv13_473_2	Amplification	13	PF3D7_1344600	lipoyl synthase (LipA)	
cnv13_478	Amplification	13	PF3D7_1348800	E1-E2 ATPase, putative	
			PF3D7_1348900	conserved Plasmodium protein, unknown function	
			PF3D7_1348900	conserved Plasmodium protein, unknown function	
			PF3D7_1349000	conserved Plasmodium protein, unknown function	
cnv13_482	Amplification	13	PF3D7_1351200	conserved Plasmodium protein, unknown function	
**cnv13_503**	Amplification	13	PF3D7_1366400	rhoptry protein RHOP148 (RHOP148)	[10]
			PF3D7_1366500	nucleoside diphosphate kinase (NDK)	
**cnv14_516**	Amplification	14	PF3D7_1412400	conserved Plasmodium protein, unknown function	[4]
cnv14_517	Amplification	14	PF3D7_1412500	actin II (ACT2)	
cnv14_541	Amplification	14	PF3D7_1431600	ATP-specific succinyl-CoA synthetase beta subunit, putative	
cnv14_543	Deletion	14	PF3D7_1432200	conserved Plasmodium protein, unknown function	
			PF3D7_1432200	conserved Plasmodium protein, unknown function	
cnv14_549	Amplification	14	PF3D7_1438800	conserved Plasmodium protein, unknown function	
			PF3D7_1438900	thioredoxin peroxidase 1 (Trx-Px1)	
			PF3D7_1439000	copper transporter	
cnv14_564	Amplification	14	PF3D7_1452700	U1 snRNA associated protein, putative	

CNV name	Type	Chromosome	Gene ID	Annotation	Previous reports
			PF3D7_1452800	conserved Plasmodium protein, unknown function	
cnv14_573	Amplification	14	PF3D7_1460700	60S ribosomal protein L27 (RPL27)	
			PF3D7_1460800	snRNA-activating protein complex subunit 3, putative	

Additional File 1. CNVs found in this study. Those marked in bold have been reported previously. CNVs showing both a deletion and amplification allele are listed separately.

References

1. Carret CK, Horrocks P, Konfortov B, Winzeler EA, Qureshi M, Newbold CI, Ivens A: Microarray-based comparative genomic analyses of the human malaria parasite Plasmodium falciparum using Affymetrix arrays. Molecular and Biochemical Parasitology 2005;144:177-186.
2. Cheeseman IH, Gomez-Escobar N, Carret CK, Ivens A, Stewart LB, Tetteh KK, Conway DJ: Gene copy number variation throughout the Plasmodium falciparum genome. BMC Genomics 2009;10:353.
3. Cheeseman IH, Miller B, Tan JC, Tan A, Nair S, Nkhoma SC, De Donato M, Rodulfo H, Dondorp A, Branch OH et al: Population structure shapes copy number variation in malaria parasites. Mol Biol Evol 2016;33:603-620.
4. Jiang H, Yi M, Mu J, Zhang L, Ivens A, Klimczak LJ, Huyen Y, Stephens RM, Su XZ: Detection of genome-wide polymorphisms in the AT-rich Plasmodium falciparum genome using a high-density microarray. BMC Genomics 2008;9:398.
5. Kidgell C, Volkman SK, Daily JP, Borevitz JO, Plouffe D, Zhou Y, Johnson JR, Le Roch KG, Sarr O, Ndir O et al: A systematic map of genetic variation in Plasmodium falciparum. PLoS Pathog 2006;2 e57.
6. Mackinnon MJ, Li J, Mok S, Kortok MM, Marsh K, Preiser PR, Bozdech Z: Comparative transcriptional and genomic analysis of Plasmodium falciparum field isolates. PLoS Pathog 2009;5:e1000644.
7. Pologe LG, Ravetch JV: Large deletions result from breakage and healing of P. falciparum chromosomes. Cell 1988;55:869-874.
8. Ribacke U, Mok BW, Wirta V, Normark J, Lundeberg J, Kironde F, Egwang TG, Nilsson P, Wahlgren M: Genome wide gene amplifications and deletions in Plasmodium falciparum. Molecular and Biochemical Parasitology 2007;155:33-44.
9. Samarakoon U, Gonzales JM, Patel JJ, Tan A, Checkley L, Ferdig MT: The landscape of inherited and de novo copy number variants in a Plasmodium falciparum genetic cross. BMC Genomics 2011;12:457.
10. Samarakoon U, Regier A, Tan A, Desany BA, Collins B, Tan JC, Emrich SJ, Ferdig MT: High-throughput 454 resequencing for allele discovery and recombination mapping in Plasmodium falciparum. BMC Genomics 2011;12:116.
11. Scherf A, Mattei D: Cloning and characterization of chromosome breakpoints of Plasmodium falciparum: breakage and new telomere formation occurs frequently and randomly in subtelomeric genes. Nucleic Acids Research 1992;20 1491-1496.
